# Ovarian tissue cryopreservation in a patient with breast cancer during pregnancy: a case report

**DOI:** 10.1186/s13048-021-00929-3

**Published:** 2021-12-12

**Authors:** Jiaojiao Cheng, Xiangyan Ruan, Juan Du, Fengyu Jin, Yanglu Li, Xiaowei Liu, Husheng Wang, Muqing Gu, Alfred O. Mueck

**Affiliations:** 1 Department of Gynecological Endocrinology, Beijing Obstetrics and Gynecology Hospital, Capital Medical University, Beijing Maternal and Child Health Care Hospital, No. 251, Yaojiayuan Road, Chaoyang District, Beijing, 100026 People’s Republic of China; 2 Department for Women’s Health, University Women’s Hospital and Research Centre for Women’s Health, University of Tuebingen, 72076 Tuebingen, Germany; 3 Department of Obstetrics, Beijing Obstetrics and Gynecology Hospital, Capital Medical University, Beijing Maternal and Child Health Care Hospital, Beijing, 100026 China

**Keywords:** Breast cancer during pregnancy, ovarian tissue cryopreservation, fertility preservation, ovarian function

## Abstract

**Background:**

Fertility preservation using ovarian tissue cryopreservation (OTC) in patients with certain diseases, especially those needing chemo- or radiotherapy, is becoming routine in various Western countries. Our hospital is the first and until now the only centre in China to use this method. The question of whether treatment of breast cancer during pregnancy (PrBC) should be similar to non-pregnant young patients with breast cancer is controversial. To our knowledge, this is the first report worldwide to use OTC as fertility preservation for PrBC.

**Case presentation:**

During the 29th week of pregnancy, a 24-year-old woman underwent needle aspiration cytology of a left breast tumour. Ultrasound and cytology revealed BI-RADS 4a grade. Oncologists recommended termination of the pregnancy. Caesarean section was performed at week 32, and ovarian tissue samples were collected for OTC to preserve fertility and ovarian endocrine function. Twenty-three ovarian cortex slices were cryopreserved. It is estimated that 13,000 follicles were cryopreserved. Breast nodules and sentinel lymph node biopsy suggested invasive micropapillary carcinoma. Neoadjuvant chemotherapy was started within 1 week after diagnosis. After six courses of neoadjuvant chemotherapy, targeted drug therapy and goserelin acetate, left mastectomy and left axillary lymph node dissection were performed. In total, 23 doses of radiotherapy, eight trastuzumab targeted therapy treatments, and 17 pertuzumab + trastuzumab double targeted therapy treatments were performed after breast cancer surgery. Until now, more than 2 years after delivery, the ovarian function still is good, and no signs of a negative impact of OTC have been observed. Goserelin acetate injections, administered every 28 days, are planned to last for the next 5 years. In addition, endocrine therapy with anastrozole was started after breast cancer surgery and also is scheduled for 5 years.

**Conclusion:**

OTC for fertility preservation in patients with PrBC does not delay breast surgery, radiotherapy or chemotherapy, which is essential for effective treatment of breast cancer. We assess this method as a promising fertility preservation method which was used here for the first time worldwide in a patient who developed breast cancer during pregnancy.

## Background

In 2020, there were 2.26 million new cases of female breast cancer worldwide, far exceeding other types of female cancers, accounting for about 24.5% of female cancers [1]. Breast cancer during pregnancy (PrBC) occurring as primary breast cancer diagnosed during pregnancy [2], accounts for about 4% of breast cancer cases in women under the age of 45 [3]. The incidence of PrBC is estimated to be about 1 in 3000 pregnancies [3]. Breast cancer during the postpartum period (PPBC), arising within 5–10 years after delivery, accounts for an estimated 35–55% of all breast cancer cases in women under 45 years of age [4]. The incidence of pregnancy-related breast cancer has increased significantly over the past 10 years [5], especially in developed countries. It may be related to the postponement of the age of first pregnancy and the continued increase in the incidence of young breast cancer [6]. With the development of early diagnosis and treatment strategies for breast cancer, the disease-free and overall survival rate of patients with breast cancer have been greatly improved [7]. However, compared with unaffected women, the fertility rate of breast cancer patients decreases significantly about 40–67% after diagnosis and treatment. More and more attention has been paid to the effects of breast cancer chemotherapy, radiotherapy, and endocrine therapy on the quality of life and fertility of breast cancer patients [8].

At present, fertility preservation strategies mainly include embryo and oocyte cryopreservation, ovarian tissue cryopreservation (OTC), in vitro maturation (IVM), and gonadotropin-releasing hormone analogue (GnRHa) therapy during chemotherapy [9]. However, to our knowledge, there are no reports about OTC in patients with PrBC. This report described performing OTC in a young patient with PrBC, which could provide a new understanding for clinicians and the first evidence for fertility preservation in patients with PrBC.

## Case presentation

The case describes a 26-year-old Chinese lady who was pregnant 2 years ago. At 15 weeks’ 15 gestation, she felt a solid lump in her left breast. Physical examination showed that the left breast mass was the size of a peanut, with milk overflow, no tenderness, and no apparent depression or bumps on the breast surface. Breast ultrasound showed mammary gland hyperplasia, and the low echo of the left breast, 12*8 mm, 11*7 mm, breast imaging reporting and data system (BI-RADS) 4a grade. The oncologist suggested re-examination after 3 months. The patient noticed that the breast mass gradually increased, so she saw a doctor again in a large grade 3A hospital. Needle aspiration cytology of the left breast tumour was performed in March 14, 2019, and cancer cells were detected. Using breast ultrasound, a solid nodule was seen in the lower left breast quadrant, 18*13 mm, clear boundary, lobulated, BI-RADS 3 grade. A solid cystic nodule, 10*8 mm, was found, and a solid area could be seen in it, range about 4*3 mm, BI-RADS 4a. Oncologists recommended that the patient should undergo termination of the pregnancy.

Caesarean section was performed at 32 weeks and 4 days of pregnancy in the Beijing Obstetrics and Gynecology Hospital, Capital Medical University, and a healthy baby boy was delivered. At the same time as the caesarean section, the ovarian tissue biopsy was taken for OTC to preserve fertility and ovarian function. Half an ovary was removed from one side, and 1/3 of the ovary from the other side. The condition of the fresh ovarian tissue was good, with a corpus luteum and rich blood vessels.

**Fig. 1:**
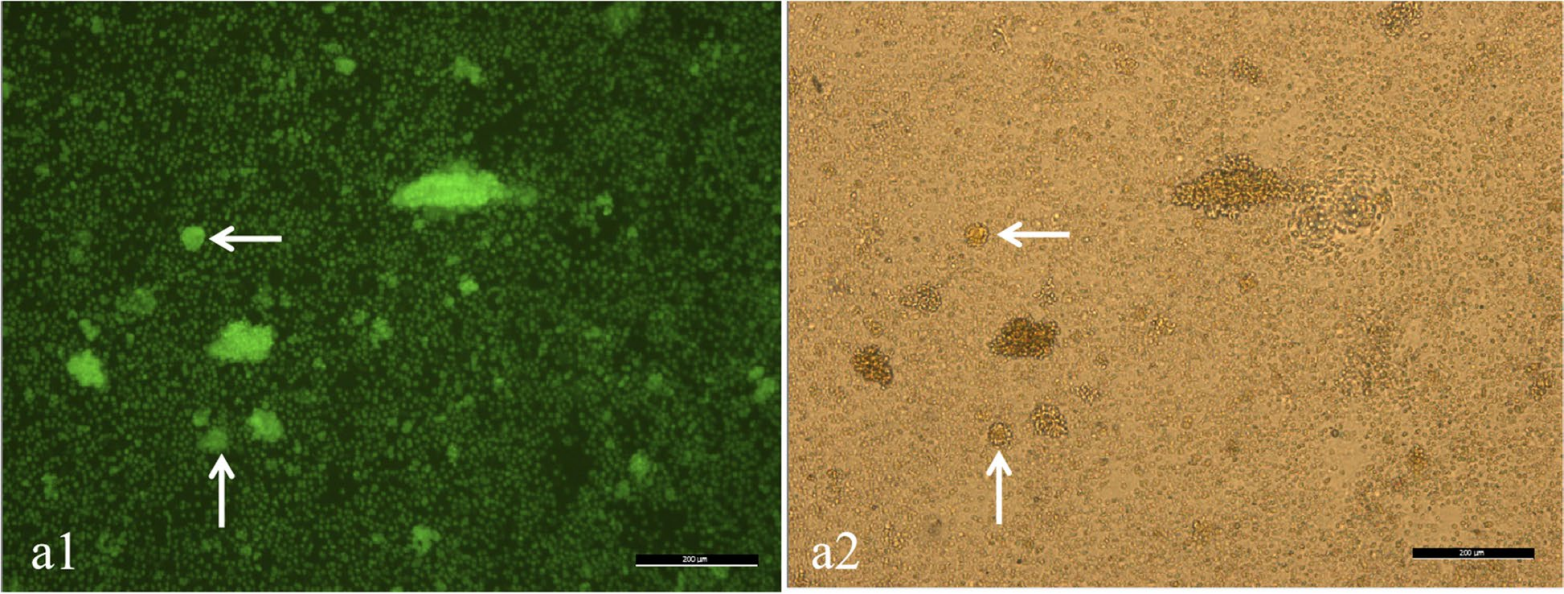
Typical pictures of follicles in the fresh ovarian cortex. a1 is the image under the fluorescence microscope, and a2 is the image under the optical microscope. The white arrow refers to the follicles. bar=200 μm

**Fig. 2:**
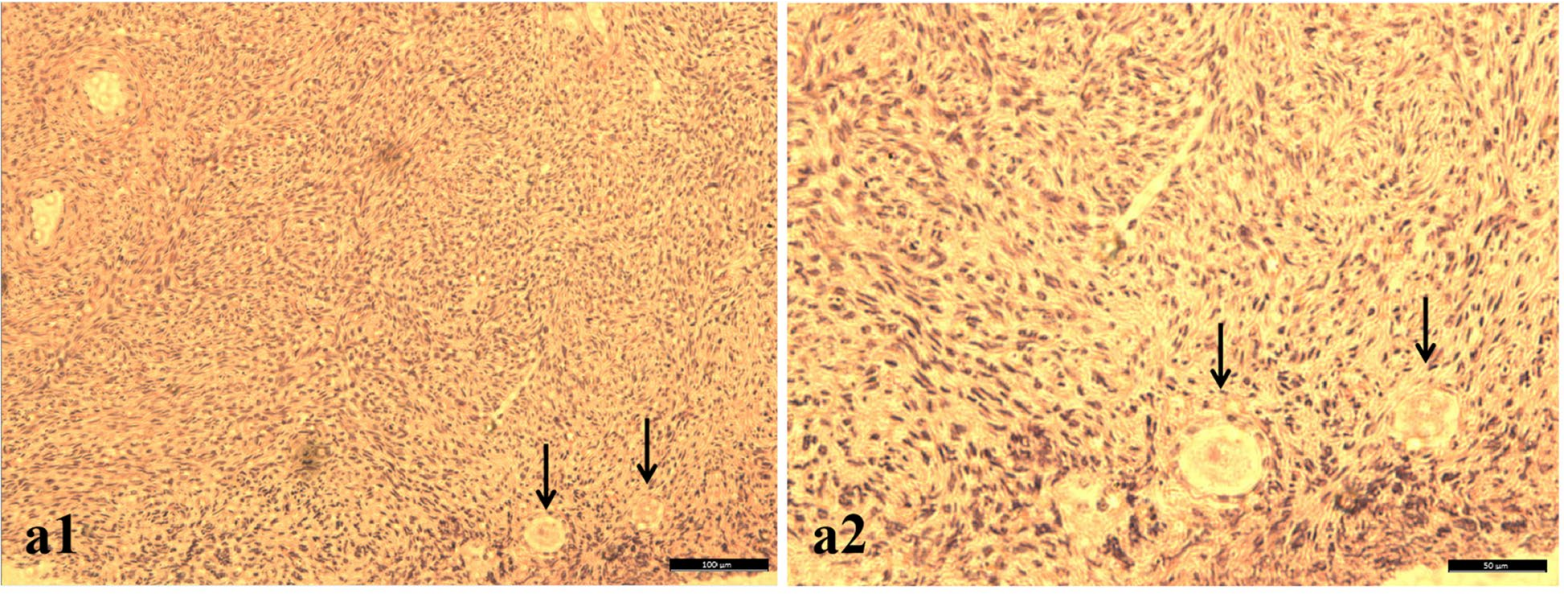
Typical HE staining images of follicles in the fresh ovarian cortex. The black arrow refers to the follicle. In a1, bar = 100 μm; in a2, bar = 50 μm

The ovarian tissue was successfully processed and slow-programmatically cryopreserved in ovarian tissue cryobank, and fresh cortex viability and morphology assessment was performed. The methods description was consistent with the previously published article [10, 11]. A total of 23 pieces of ovarian cortex were frozen. The number of follicles in a round cortical piece of 2 mm is about 45, and it is estimated that 13,000 follicles were frozen for this patient. The images of follicular viability are shown in Fig. 1, and the images of HE staining in the cortex are shown in Fig. 2.

A biopsy of the left breast tumour was performed on April 18, 2019. Pathological findings showed (breast tissue) invasive micropapillary carcinoma of the breast, immunohistochemical results: estrogen receptor (ER) (weak-medium positive, 10%), progesterone receptor (PR) (−), androgen receptor (AR) (weak-medium positive, 80%), human epidermal growth factor receptor 2 (HER-2) (3 +). Ki-67 (index 30%), p53 (+), cytokeratin 14 (CK14) (−), D2–40 (−), epithelial membrane antigen (EMA) (+), CK5/6 (−), epidermal growth factor receptor (EGFR) (−), synaptophysin (Syn) (−), chromogranin A (CgA) (−). A left axillary sentinel lymph node biopsy was performed on April 24, 2019. Pathology showed that the metastatic carcinoma of the lymph node (left sentinel) (2 positives in 3) and the isolated tumour cells could be seen in the capsule of the other lymph node.

Neoadjuvant chemotherapy was administered prior to breast surgery. The first chemotherapy regimen was docetaxel 120 mg, epirubicin 110 mg, and cyclophosphamide 900 mg, q3w. Injections of 3.6 mg goserelin acetate every 28 days were given starting after the first course of chemotherapy, planned to continue for 5 years. No menstruation occurred after the start of chemotherapy. According to the immunohistochemistry results, the 2nd-6th chemotherapy regimen was adjusted as follows: docetaxel 110 mg and carboplatin 500 mg. Targeted therapy was administered with trastuzumab, the first dose was 17 ml, which was then changed to 13 ml. Six doses of targeted therapy were administered before breast surgery.

Ultrasound examination of breast and axillary lymph nodes before breast surgery showed that low echo was seen at the edge of the gland in the direction of 6–7 o’clock in the left breast, 19*16*8 mm. The boundary was unclear, and a dotted strong echo was diffused in the left breast, BI-RADS 6.

Simple left mastectomy (preserving nipple-areola) and left axillary lymph node dissection were performed on September 10, 2019. Left breast reconstruction + acellular allogenic dermis implantation + dilator implantation was performed after breast surgery. Pathological report: a few invasive carcinomas in the (left) breast (the largest 7 mm), and a vascular tumour thrombus, the bottom cutting edge not special, lymph nodes showing chronic inflammation (left axilla 0/18). Immunohistochemical results showed: ER (weak positive, 10%), PR (−), AR (weak positive, 90%), Her-2 (3 +), Ki-67 (index25%), p53 (scattered +), EGFR (−), CK14 (−), CK5/6 (−), p63 (−), CgA (−), Syn (−), EMA (+).

After breast surgery, fixed-field intensity-modulated radiation therapy was performed with a 6MV-X line. 95% of the plan clinical tumour volume included left upper and lower clavicle, chest wall, dose 46Gy/23 times (2Gy/f), 5 f / w, filler bolus 0.5 cm.

Fourteen courses of trastuzumab were administered after surgery, followed by about 17 courses of pertuzumab + trastuzumab 13 ml double targeted therapy for 1 year. After breast surgery, goserelin acetate was continued every 28 days, and is planned to last for 5 years. Endocrine therapy using anastrozole, a potent aromatase inhibitor, is also scheduled to last for 5 years.

The serum hormone levels before OTC and 1 year, 1.5 years, 2 years, and 2.2 years after OTC are shown in Table 1. At present, it seems that the AMH level of the patients is in the normal range, and the levels of FSH, LH, and E2 are all at a low level.

**Table 1:** Hormone levels of before and after cryopreservation

	2019.04.08 (before OTC)	2020.05.08 (1 year after OTC)	2020.10.12 (1.5 years after OTC)	2021.03.17 (2 years after OTC)	2021.06.18 (2.2 years after OTC)
AMH (ng/ml)	2.73	1.18	1.3	2.54	3.70
FSH (IU/L)	0	4.05	6.08	3.66	4.64
LH (IU/L)	0	1.46	1.31	1.03	0.39
E2 (pg/ml)	15,000	11.8	11.8	33.67	19.37

*AMH* anti-müllerian hormone, *FSH* follicle-stimulating hormone, *LH* luteinizing hormone, *E2* estradiol, *OTC* ovarian tissue cryopreservation

## Discussion and Conclusion

A 26-year-old woman in the third trimester of pregnancy was diagnosed with PrBC more than 2 years ago and underwent caesarean section at 32 weeks and 4 days to terminate the pregnancy. Subsequently, the patient underwent chemotherapy, radiotherapy, targeted therapy, breast cancer surgery, and long-term endocrine therapy. During caesarean section, ovarian tissue samples were collected and OTC was performed to preserve fertility and ovarian function, without additional ovarian tissue biopsy surgery. This technique for fertility preservation avoids ovarian hyper-stimulation, and there is no need to delay follow-up anti-cancer treatment. For this patient, the risk of premature ovarian insufficiency (POI) in the future is high, but her survival rate is also very high. Only one child was born, and the patient still is young and wishes to preserve ovarian function and fertility.

PrBC should be regarded as an independent entity to breast cancer occurring during the postpartum period (PPBC). The treatment of PrBC is individualized according to gestational age and considers the safety of the foetus. The treatment of PPBC does not need to consider these issues. The histopathological and immunohistochemical results of tumours in PrBC patients seem to be similar to those in young non-pregnant breast cancer patients. High estrogen and progesterone levels during pregnancy may stimulate the proliferation of breast cancer cells in PrBC patients. However, the prognosis does not seem to differ from non-pregnant patients of the same age and stage [3, 4]. As with the patient in this study, the current outcome is good. Therefore, the biological characteristics of the tumour are more likely to be determined by the age at the time of diagnosis than by pregnancy.

In 2013, the American Society of Clinical Oncology conducted a comparative study of 311 patients with PrBC and 865 patients with non-pregnancy-related breast cancer [12]. It was found that the overall survival rate of patients with PrBC was similar to that of patients with non-pregnancy-related breast cancer. This information is essential when the PrBC patient is consulted and supports the start of treatment while pregnancy can continue. For our case, the pregnancy was terminated after confirmation of the diagnosis according to the oncologists’ recommendations, i.e. anti-cancer treatment was not started during pregnancy.

The 5-year relative survival rate of breast cancer patients is about 90% [13]. More than 50% of young women with breast cancer wish to become pregnant after treatment [14]. It is reported that their chances of pregnancy are 40–67% lower than that of the general population [15]. Some studies have reported that the live birth rate for breast cancer patients after treatment is less than 5% [16, 17]. Recently, a large sample study showed that in breast cancer patients with and without fertility preservation, the cumulative incidence of live births after 5 years of breast cancer diagnosis was 19.4 and 8.6%, respectively, and 40.7 and 15.8% 10 years later [18]. The research on pregnancy safety after breast cancer treatment is complex, and randomized controlled trials are impossible, so the evidence to guide clinical practice is limited.

The best time to conceive after the diagnosis of breast cancer is still inconclusive. The main concern is the recurrence of cancer and the interruption of endocrine therapy. ER-negative patients should be delayed for 2–3 years according to the prognosis. Positive patients can discuss whether to discontinue endocrine treatment after 3 years, but patients must be informed of the lack of data support [19]. One large meta-analysis [20] found that post-breast cancer pregnancy had no adverse effect on survival. Women who became pregnant after breast cancer had even higher survival rates than non-pregnant breast cancer patients [21].

Patients with PrBC received chemotherapy during pregnancy and post-natal chemotherapy, and an observational study reported no difference in survival rate [22]. Chemotherapy during pregnancy is generally carried out in the third trimester of pregnancy, and there is no increase in the rate of congenital malformations. The available data confirm that the mother and foetus are safe whilst using breast cancer treatment during pregnancy [22]. Because preterm delivery is closely related to adverse events, full-term delivery seems to be the most important.

The degree of ovarian function damage caused by breast cancer chemotherapy is related to the patient’s age, chemotherapy type, dose, and duration [23]. Among the commonly used chemotherapeutic drugs, alkylating agents have the strongest gonadal toxicity, followed by platinum, paclitaxel, anthracycline, and so on. Some patients may have temporary or permanent amenorrhea during chemotherapy. 40–60% of women under 40 years old will have amenorrhea, and more than 80% of women over 40 years old will have amenorrhea. Although some patients’ menstruation can recover after chemotherapy, ovarian function is still impaired. This suggests that menstruation does not necessarily mean giving birth [24, 25]. It is recommended that fertility preservation strategies should be taken as far as possible for patients who still have fertility wishes in the future before the start of chemotherapy [26].

Radiotherapy is an essential part of comprehensive breast cancer treatment. It is an important measure to reduce the recurrence and prolong the survival of patients undergoing breast-conserving surgery and high-risk mastectomy [27]. In breast cancer patients receiving standard whole breast radiotherapy, 2.1–7.6 cGy (1Gy = 100 cGy) reaches the uterus and ovaries through the internal scattering of 50Gy radiation dose to the breast [28]. Radiotherapy is not recommended for PrBC but can be chosen according to the condition after stopping breastfeeding at the end of pregnancy [29].

Endocrine therapy refers to drugs to block the promoting effect of sex hormones on breast cancer cells according to the expression of ER and PR in breast cancer tissue [30]. Anastrozole is a potent, selective aromatase inhibitor of triazole, blocking estrogen biosynthesis by inhibiting aromatase [31]. Estrogen is the main factor that stimulates the growth of breast cancer cells. Although endocrine therapy has no reproductive toxicity, endocrine treatment lasts for 5–10 years, and the ovarian function of patients continues to decrease with age. Therefore, breast cancer patients with fertility needs are recommended fertility preservation before endocrine therapy [7].

Embryo cryopreservation is the most widely used and technically perfect fertility preservation strategy in the clinic, suitable for married women after puberty [32]. Two aspects are worth paying attention to 1) due to a series of procedures such as ovulation stimulation, in vitro fertilization, and embryo cryopreservation, the treatment of breast cancer may be delayed for about 2 weeks; 2) the hyper-physiological dose of estrogen caused by ovulation stimulation maybe promote the development of breast cancer. There has been a report [33] of fertility preservation with random-start controlled ovarian stimulation and embryo cryopreservation for early pregnancy-associated breast cancer.

Oocyte cryopreservation also is suitable for unmarried women after puberty. It is recommended that women < 38 years old freeze 15–20 MII stage oocytes, the chance of at least one live birth is 70–80%. For 38–40-year-old women, if 25–30 MII stage oocytes are frozen, the chance of at least one live birth is 65–75% [34]. IVM can reduce ovarian stimulation, avoid a high estrogen state, and be combined with OTC [35]. Although it is estimated that more than 5000 babies have been born through IVM technology worldwide [36], IVM is still considered an experimental technology by the American Society of Reproductive Medicine (ASRM) [37].

The OTC technique requires minimally invasive surgery to remove ovarian tissue before gonadotoxicity treatment, without the need for ovarian stimulation to obtain oocytes. It is the only fertility preservation method for patients who cannot delay anti-cancer treatment [38, 39]. More than 200 babies have been born worldwide after OTC and transplantation [40], and in 2019 the ASRM stated that this technique is no longer experimental [41]. The ovarian tissue cryobank of Beijing Obstetrics and Gynecology Hospital, the first and until now the only one in China [42], has successfully cryopreserved more than 370 cases of ovarian tissue up to now, of which 10.8% are breast cancer patients. A total of 10 cases of ovarian tissue were transplanted [11, 43], including 1 case of breast cancer with negative ER, PR, and HER2 (3 +). The ovarian function was recovered after transplantation. One patient with myelodysplastic syndrome (MDS) successfully became pregnant naturally [44] after ovarian transplantation and a healthy baby girl was born (article under review). The timing of ovarian tissue transplantation in this breast cancer patient should be discussed with a breast cancer specialist. The main indicators are as follows [42]: 1) multidisciplinary cooperation is needed; 2) the patient is disease-free and endocrine therapy at least 2 years or can be stopped and stopped at least 3–6 months; 3) ovarian function failure; 4) there is a desire for fertility.

Breast cancer is one of the most common indications for OTC and transplantation. In five major centres in Europe, of 285 transplant patients, 96 were breast cancer patients and 7 had relapsed (7.3%) [45]. Breast cancer itself is a known disease with a risk of recurrence [46]. At the time of breast cancer diagnosis, young age is associated with an increased risk of recurrence [47], while patients who receive OTC are usually very young and almost all are under the age of 40. All relapses were dependent of the primary disease and had nothing to do with ovarian tissue transplantation. All relapses were far away from the transplant site, and most of them were close to the location of the primary cancer. The recurrence rate of 7.3% is similar to that of breast cancer women under 40 years old observed in the literature, with a local recurrence rate of 10% [48] and a 10-year recurrence rate of 4–8.7% [46]. Regarding transplanting ovarian tissue from breast cancer patients, it should be kept in mind that they are still cancer patients in remission.

## Conclusion

Ovarian tissue cryopreservation for fertility preservation in patients with PrBC does not delay breast surgery, radiotherapy, or chemotherapy which is essential for effective treatment of breast cancer. Until now, more than 2 years after delivery, we do not see any signs of a negative impact of performing OTC at the same time as caesarean section, and subsequently receiving chemotherapy, radiotherapy, targeted therapy, breast cancer surgery, and endocrine therapy. At present, the patient’s ovarian function is still good. Although long-term results and especially the results after retransplantation of ovarian tissue still are missing, we assess this method, which to date has only been performed in our hospital in China, as a promising fertility preservation method for patients who develop breast cancer during pregnancy.

## Data Availability

All the generated data are included in this article.

## Abbreviations

OTC: Ovarian tissue cryopreservation
POI: Premature ovarian insufficiency
PrBC: Breast cancer occurs during pregnancy
PPBC: Breast cancer occurs during the postpartum period
IVM: In vitro maturation
GnRHa: Gonadotropin-releasing hormone analogue
BI-RADS: Breast imaging reporting and data system
ER: Estrogen receptor
PR: Progesterone receptor
AR: Androgen receptor
HER-2: Human epidermal growth factor receptor 2
EMA: Epithelial membrane antigen
EGFR: Epidermal growth factor receptor
Syn: Synaptophysin
CgA: Chromogranin A
ASRM: American society of reproductive medicine
FSH: Follicle stimulating hormone
LH: Luteinizing hormone
AMH: Anti-müllerian hormone
E2: Estradiol
MDS: Myelodysplastic syndrome
